# Case Report: Suspected intestinal perforation caused by accidental ingestion of magnet

**DOI:** 10.3389/fsurg.2025.1583175

**Published:** 2025-08-07

**Authors:** Mengyu Ke, Jun Yang

**Affiliations:** ^1^ School of Medicine, Jianghan University, Wuhan, China; ^2^ Wuhan Children’s Hospital (Wuhan Maternal and Child Healthcare Hospital), Tongji Medical College, Huazhong University of Science & Technology, Wuhan, China

**Keywords:** foreign body, ingestion, perforation, magnets, case report

## Abstract

**Introduction:**

Children frequently swallow foreign objects, and ingesting magnets on occasion can cause major problems because they are composed of unique materials that draw to one another in the intestinal wall. As a result, there is a significant chance of intestinal wall necrosis, intestinal perforation, and fistula formation. On the basis of the history of magnet ingestion, clinical signs like nausea and vomiting, and x-ray imaging, it is easy to diagnose magnet-induced complications. However, there was no foreign body shadow in the x-ray, and some intermittent swallowing of magnets in different intestinal tubes attracted one another, causing intestinal perforation and subsequent discharge along with the feces.

**Case presentation:**

We describe the case of an 8-year-old girl who had been consuming two magnets for six days when she arrived at our hospital's emergency room with sporadic stomach ache and vomiting for two days. The youngster had a history of vomiting yellow-green bile, and a physical examination revealed a noticeable intestinal pattern with noticeable periumbilical discomfort. An abdominopelvic CT scan indicated intestinal obstruction, while an abdominal x-ray indicated minimal distension of the intestinal tube and a fluid level. No obvious foreign body shadow was seen in the abdominal cavity. Laparoscopic exploration revealed two perforations, one in the colon and one in the small bowel; no magnets were found intraoperatively. Intraoperative abdominal radiographs suggested loss of signs of limited intestinal distension and no foreign body was seen. Considering the absence of foreign bodies observed during the operation and the patency of the intestine postoperatively, it is reasonably inferred that the magnet was excreted via feces after causing intestinal perforation. The patient's condition was stable after the operation and she was discharged from the hospital after 9 days.

**Conclusion:**

This case emphasizes the necessity of maintaining clinical vigilance in pediatric patients with suspected magnet ingestion, even when imaging fails to identify foreign bodies. Notably, the absence of intraoperatively retrieved magnets does not preclude magnet-induced injuries, and early surgical exploration may contribute to improved clinical outcomes.

## Introduction

Foreign body ingestion in children is not uncommon among childhood accidental injuries.80%–90% of ingested foreign bodies can pass through the gastrointestinal tract on their own, and less than 1% can cause serious complications requiring surgical intervention (1–3). Among the various foreign bodies swallowed, magnet ingestion is a very serious problem, especially when multiple magnets are ingested intermittently, they can attract each other on the intestinal wall leading to serious consequences. It is estimated that there are 3.06 cases of magnet misuse per 100,000 children per year (4), and this figure continues to grow each year with the increase in modern magnetic toys. When one small magnet is misused, there are frequently no visible clinical indications, but when two or more magnets are consumed sporadically, substantial injury results. The intestinal wall is drawn in by the strong attraction between these magnets when they are in magnetic range of one another. This causes pressure necrosis, which can result in tissue ischemia, perforation and peritonitis, fistula formation, or intestinal obstruction (2, 5, 6). Diagnosis of magnet-induced complications typically relies on ingestion history, clinical symptoms (e.g., nausea, vomiting), and imaging. However, challenges arise when magnets are excreted before presentation, leaving no radiological evidence. This report describes a case of suspected intestinal perforation secondary to magnet ingestion, where no magnets were identified intraoperatively, to highlight the diagnostic complexities in such scenarios.

## Case presentation

**Figure 1:**
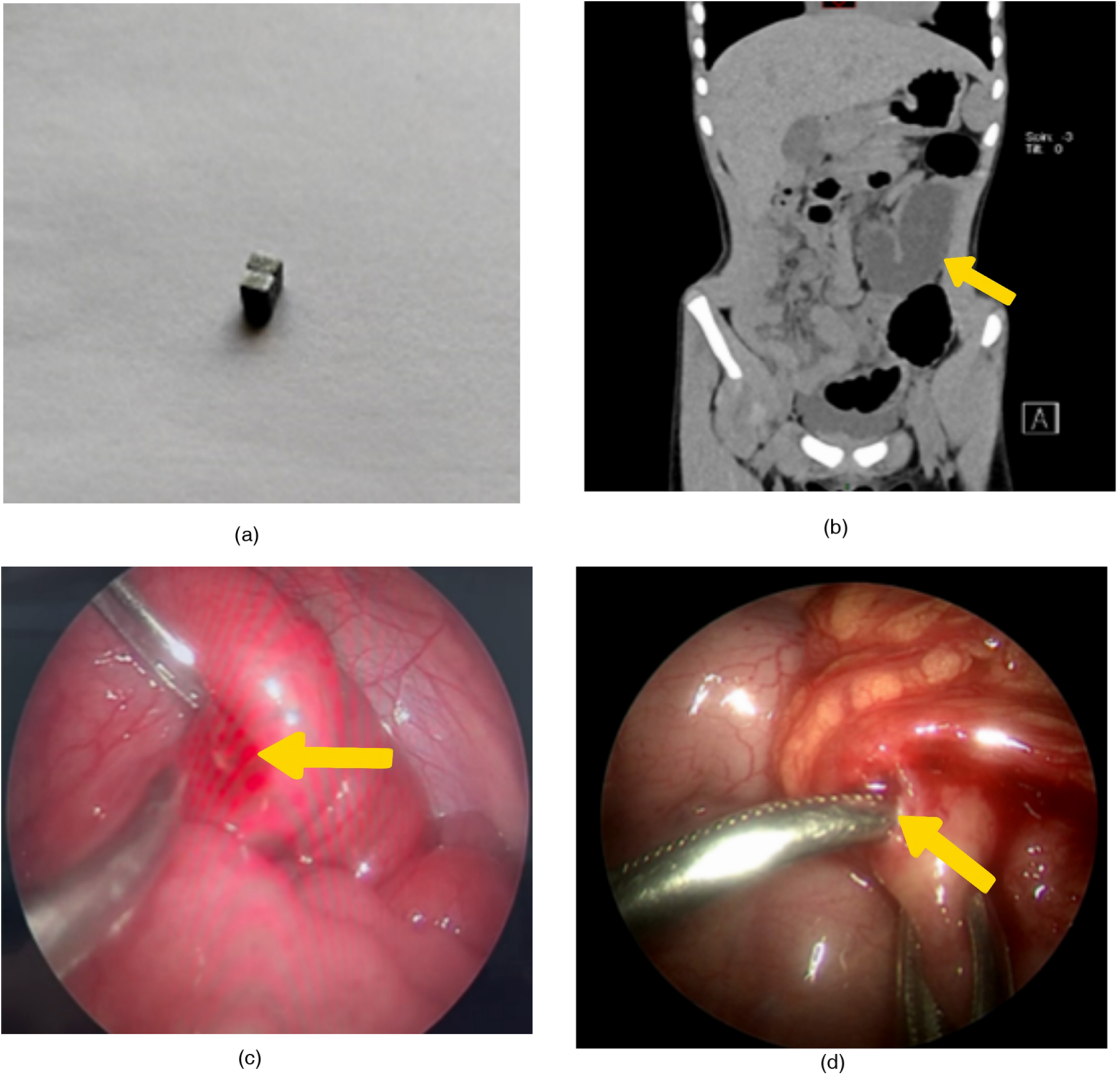
**(a)** the mother provided two homotypic square magnets, which were identical to the magnet the child had been playing with but not swallowed. **(b)** The non-contrast CT scan shows marked dilation of the proximal small intestine and complete emptiness of the distal bowel. Intestinal obstruction is considered. **(c)** Enteric perforation located 60 cm from the ileocecal valve in the ileum. **(d)** Colonic perforation located in the mid-transverse colon.

An 8-year-old girl was admitted to our emergency room 6 days ago due to ingestion of two magnets for 6 days and intermittent abdominal pain and vomiting for 2 days. Physical examination revealed a mildly distressed face, marked periumbilical tenderness, no rebound pain, and a visible bowel pattern, and a history of vomiting of yellowish-green bile, with no other symptoms. According to both the child's mother and the youngster herself, two tiny square magnets that were roughly 0.2 × 0.3 cm^2^ were ingested non-simultaneously with a 2-hour interval (Figure 1a). There were no notable abnormalities in the family history, allergy history, or psychosocial history.

The child's vital signs were within normal ranges upon evaluation. The neutrophils were 92.9%, and the WBC and ultrasensitive C-reactive protein were also within normal limits. There was no visible opaque foreign body shadow in the belly, however abdominal radiographs indicated modest distention of the bowel with fluid level. There was no visible high-density foreign body shadow in the stomach or intestinal lumen, and the abdominal and pelvic CT scan revealed localized small bowel dilatation, gas and fluid accumulation, unclear intestinal gap, highly dilated proximal small bowel, and completely empty distal bowel, all of which were thought to be intestinal obstructions (Figure 1b). The above auxiliary examinations did not reveal any foreign body shadow in the abdominal cavity or signs of intestinal perforation, but in combination with the history of non-simultaneous ingestion of magnets, obvious abdominal pressure and pain, visible intestinal pattern, and vomiting of yellowish-green bile, and in combination with CT, the child was considered to have intestinal obstruction, and was then subjected to laparoscopic exploration for diagnosis and further treatment.

At the time of exploration, the jejuno-ileal bowel was highly dilated and the distal bowel was empty. The small intestine was explored retrogradely from the ileocecal region, and one perforation of the ileum was seen at 60 cm from the ileocecal region, with a size of about 0.8 × 0.8 cm (Figure 1c), the margin showed congestion with granulation tissue formation and the proximal part was continued to be explored, but no perforation site was found. After exploring the colon from the distal portion of the ileum, the omental covering was visible at the middle section of the transverse colon. The local omentum was red and swollen, and upon lifting it, a perforation measuring roughly 0.8 × 0.8 cm was visible (Figure 1d). Despite further exploration, no clear signs of an inflammatory reaction or perforation site were discovered. Additionally, two intestinal perforations were fixed. No foreign body was observed, and intraoperative abdominal radiographs indicated that the indications of limited intestinal distension had disappeared.

On the fourth postoperative day, the patient started to pass gas, the abdominal drain was removed, and he could drink water. On the 9th day of admission, the patient's condition remained stable, and a follow-up gastrointestinal ultrasound showed a small amount of abdominal effusion without complications, leading to discharge. Two months after surgery, repeated gastrointestinal ultrasound revealed no obvious abdominal abnormalities.

## Discussion

The prognosis is generally better if the magnet ingestion can be clearly diagnosed at an early stage and the child is closely monitored and followed up until the magnet is expelled from the body and no other clinical symptoms are observed. In children, intestinal perforation due to magnet ingestion is common and can cause varying degrees of complications depending on the number of magnets ingested and the length of the disease. However, most of the children come to the clinic only after the foreign body has been ingested for a long time because the clinical symptoms are not prominent enough, and the abdominal signs and imaging may not show any obvious serious complications, which may easily lead to missed diagnosis of intestinal perforation and other complications, which may lead to serious consequences.

When children swallow smooth foreign objects like steel balls and buckyballs, they can be expelled if they go through the esophagus without any problems. However, when foreign objects with unique structures and characteristics, like magnets, are swallowed, they can cause complications like obstruction, perforation, and enterocutaneous fistulae, which happen in the small intestine and pharynx (7). These issues frequently arise because of the attraction between magnets in various intestinal segments, which puts pressure on the intestinal wall and causes tissue ischemia necrosis.

The majority of magnet ingestion diagnoses are made after problems have arisen (8, 9). Abdominal pain, vomiting, and fever are common clinical signs in patients who have intermittently consumed several magnets. Surgery and/or conservative treatment have been used to treat magnet ingestion in children (10–12). Studies have indicated that just 1% of cases require surgery to treat associated problems. Conservative treatment is often recommended for single magnet ingestion (13). Cesarean sections, laparoscopic procedures, and endoscopic resections are examples of surgical treatments. If more than one magnet is swallowed and does not pass through the pylorus, endoscopic resection ought to be done right away. In cases where the magnet crosses the pylorus and the patient is asymptomatic, surgery is also required (14). This is because when the magnets are located in different intestinal segments the magnetic forces attract each other and compress the intestinal wall, which can easily lead to perforation.

Because they are positioned in separate intestinal sites and attract one another, several magnets adhering together can be mistakenly perceived as a single entity in a single intestinal lumen (10), which can result in intestinal perforation or fistula (15). In order to assess the severity of the disease in conjunction with clinical manifestations and auxiliary examinations, we clinicians should inquire in-depth about the type and quantity of magnets consumed by these children, as well as the swallowing sequence and other medical history. However, while CT and x-ray examinations can assist in identifying intestinal perforation and foreign body ingestion, they are not definitive methods for diagnosis, and we must also take into account the possibility that the foreign body was evacuated from the body. This demonstrates that a thorough medical history is necessary for both diagnosis and treatment. Undoubtedly, the aforementioned tests can optimize our function in verifying the diagnosis and course of treatment.

This case highlights the challenge of diagnosing magnet-induced intestinal injury when foreign bodies are absent on imaging or intraoperatively. The diagnosis of suspected magnet-induced intestinal perforation, despite the absence of intra-operative magnet visualization, was established on the basis of the following criteria: First, the perforation located between the ileum and transverse colon represented a characteristic manifestation of ingested magnets (15). Second, there was a well-documented clinical history of magnet ingestion (as reported by the child and his mother). Third, imaging studies revealed intestinal obstruction with segmental bowel wall thickening. Fourth, alternative etiologies of perforation—including infectious enteritis, intussusception, Meckel's diverticulum, and trauma—were systematically excluded. This exclusion was supported by: normal leukocyte count and negative stool culture results excluding infectious etiology; absence of the pathognomonic “target sign” on CT scan ruling out intussusception; intra-operative exploration showing no diverticulum to exclude Meckel's diverticulum; and lack of traumatic history. Notably, the absence of retained magnets distinguishes this case from previous reports (10, 15), where magnets were identified intraoperatively. This suggests magnets may cause perforation and be excreted before surgical intervention, complicating diagnosis.

Although the imaging shows no obvious foreign body shadow, which has affected the judgment of the condition to some extent. Considering that the child has a history of magnet ingestion, accompanied by severe abdominal pressure, pain, and has vomited yellow - green bile, the possibility of intestinal obstruction is highly suspected. Since the imaging also indicates intestinal obstruction, after comprehensive evaluation, it is determined to perform laparoscopy to clarify the diagnosis and conduct treatment. Intraoperative findings included intestinal perforation and adhesive obstruction. The marginal congestion and granulation tissue at the ileal perforation site suggested chronic pressure necrosis. The transverse colon perforation was covered by omental adhesions, and the absence of free intraperitoneal air was consistent with localized fistula formation. At the same time, we guessed that there was pulling during intraoperative exploration of the bowel, which led to the separation of the perforated adhesion site, so that no obvious stenosis and dilatation of the bowel were seen intraoperatively, but the perforation site could be found. Fortunately, due to the presence of adhesions, only a small amount of fecal matter was spilled from the perforation, and mild infection occurred. Postoperatively, the child was stable and was discharged from the hospital. However, this case has limitations: the lack of direct evidence (such as intraoperative identification of magnets or pathological confirmation of magnetic particles) to confirm that the perforation was caused by magnets. Therefore, interpretations should be made with caution, and further studies on such rare manifestations are warranted. In summary, for pediatric patients with a history of magnet ingestion accompanied by symptoms (e.g., bilious vomiting, localized tenderness), laparoscopic exploration should be considered as early as possible even in the presence of negative imaging findings, so as to avoid delayed complications such as peritoneal infection. To prevent the recurrence of such events, caregivers should be advised to provide health education on children's eating habits and try to accompany play to avoid swallowing them.

## Data Availability

The original contributions presented in the study are included in the article/Supplementary Material, further inquiries can be directed to the corresponding author.
